# The effects of raspberry consumption on lipid profile and blood pressure in adults: A systematic review and meta‐analysis

**DOI:** 10.1002/fsn3.3940

**Published:** 2024-01-16

**Authors:** Mostafa Shahraki Jazinaki, Hossein Bahari, Mohammad Rashidmayvan, Seyyed Mostafa Arabi, Iman Rahnama, Mahsa Malekahmadi

**Affiliations:** ^1^ Department of Nutrition, Faculty of Medicine Mashhad University of Medical Sciences Mashhad Iran; ^2^ Student Research Committee Mashhad University of Medical Sciences Mashhad Iran; ^3^ Transplant Research Center, Clinical Research Institute, Mashhad University of Medical Sciences Mashhad Iran; ^4^ Department of Nutrition, Food Sciences and Clinical Biochemistry, School of Medicine, Social Determinants of Health Research Center Gonabad University of Medical Science Gonabad Iran; ^5^ Noncommunicable Diseases Research Center Neyshabur University of Medical Sciences Neyshabur Iran; ^6^ Healthy Ageing Research Centre Neyshabur University of Medical Sciences Neyshabur Iran; ^7^ Binaloud Institute of Higher Education Mashhad Iran; ^8^ Imam Khomeini Hospital Complex Tehran University of Medical Sciences Tehran Iran

**Keywords:** blood pressure, lipid profile, meta‐analysis, raspberry, systematic review

## Abstract

Research into the effects of raspberry on blood pressure and lipid profiles is inconclusive. This meta‐analysis was aimed to determine whether raspberry has beneficial effects in clinical practice and to what extent these effects are associated with blood pressure and lipid profiles. A systematic literature search up to September 2023 was completed in PubMed/Medline, Scopus, and Web of Science, to identify eligible RCTs. Heterogeneity tests of the selected trials were performed using the *I* ^2^ statistic. Random effects models were evaluated based on the heterogeneity tests, and pooled data were determined as weighted mean differences with a 95% confidence interval. Eleven randomized controlled trials (with 13 arms) were eligible for this meta‐analysis. Our findings revealed that Raspberry consumption had no significant effects on the blood pressure and lipid profile markers, including systolic blood pressure (SBP) (WMD, −0.37 mm Hg; 95%CI: −2.19 to 1.44; *p* = .68), diastolic blood pressure (DBP) (WMD, −2.14 mm Hg; 95%CI: −4.27 to 0.00; *p* = .05), total cholesterol (TC) (WMD, −6.83 mg/dL; 95%CI: −15.11 to 1.44; *p* = .10), triglycerides (TG) (WMD, −5.19 mg/dL: 95%CI: −11.76 to 1.37; *p* = .12), low‐density lipoprotein‐cholesterol (LDL‐C) (WMD, −5.19 mg/dL; 95%CI: −11.58 to 1.18; *p* = .11), and high‐density lipoprotein‐cholesterol (HDL‐C) (WMD, 0.82 mg/dL; 95%CI: −1.67 to 3.32; *p* = .51), compared to control groups. Subgroup analysis showed that raspberry consumption significantly decreased total cholesterol and LDL‐C levels in people with elevated TC levels, metabolic syndrome, and andropause symptoms, as well as those older than 35, while the consumption of raspberries led to a significant increase in HDL‐C levels in females, obese, under 35, and healthy individuals. Raspberry can improve lipid profile and blood pressure, but it is important to keep in mind that further research is necessary to fully understand the exact mechanism of action and a definite conclusion in this regard.

## Graphical abstract

This meta‐analysis revealed that raspberry consumption did not lead to significant changes in lipid profile and blood pressure levels compared to control groups. However, it seems that more RCTs with a larger sample size and higher sensitivity are required to draw a firm conclusion.

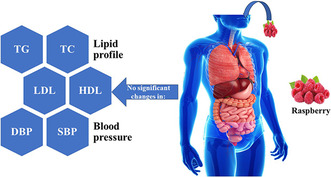

## 1 INTRODUCTION

Hypertension and dyslipidemia are the principal risk factors for cardiovascular diseases (CVD) (Ke et al., 2018). Despite extensive research, diagnostic tools, and effective treatment, they are the leading causes of CVD mortality and disability‐adjusted life years worldwide (Forouzanfar et al., 2016). When hypertension and dyslipidemia coexist, their combined negative effect on the cardiovascular system is greater than the sum of their separate effects (Borghi, 2002; Ke et al., 2018). Interestingly, it has been demonstrated that CVD risk can be significantly reduced using therapeutic approaches that target both hypertension and dyslipidemia (Borghi et al., 2022; Schwalm et al., 2016). This bidirectional synergistic effect indicates endothelial dysfunction plays a crucial role in the development of both hypertension and dyslipidemia (Dąbrowska & Narkiewicz, 2023).

In contrast to the noticeable negative effects of pharmaceutical treatments, nutritional interventions for disease management are often well‐tolerated (McInnes, 2005). Dietary Approaches to Stop Hypertension (DASH) diet, losing weight, and getting more exercise are the main ways to treat high blood pressure. Numerous randomized controlled trials on humans have shown that certain foods reduce blood pressure and protect the heart (Mansour et al., 2015; Wightman & Heuberger, 2015). Raspberry was among the foods that gained attention. Raspberry bioactive compounds like polyphenols, anthocyanins, and dietary fiber (Nile & Park, 2014) have been proposed to impact lipid metabolism, With the potential to improve lipid profile through modulating enzymes involved in lipid digestion, absorption, and synthesis (Teng et al., 2017), raspberry has been found to be effective in treating dyslipidemia, hypertension, diabetes, and obesity, and it also has anti‐inflammatory and antioxidant properties (Harasym & Oledzki, 2014). The prevention and treatment of atherosclerosis and the improvement of endothelial cell function have both been linked to raspberry. Raspberry extract was shown to reduce blood pressure in a rat model of essential hypertension (Lee et al., 2014). Raspberry's impact on blood pressure has only been evaluated in a small number of human‐controlled studies (Basu et al., 2010; Erlund et al., 2008). Blood pressure was successfully reduced in a clinical trial using a mixture of berries that included black raspberry. Eight weeks after starting treatment, prehypertensive patients who consumed black raspberries had significantly reduced 24‐hour blood pressure (Jeong, Hong, et al., 2016; Jeong, Kim, et al., 2016). The use of raspberries has been shown in one human clinical trial to improve vascular endothelial function and reduce total cholesterol and inflammatory cytokines levels in patients with metabolic syndrome (Myung et al., 2016). Black raspberry has been shown to improve vascular function, lipid profiles, and blood pressure in a few studies (Ash et al., 2011; McAnulty et al., 2014).

While the aforementioned points do raise the possibility of positive outcomes, research into the effects of raspberry on blood pressure and lipid profiles remains inconclusive. Accordingly, this meta‐analysis was conducted to determine whether raspberry has beneficial effects in clinical practice and to what extent these effects are associated with blood pressure and lipid profiles.

## 2 METHODS

All stages of designing and conducting this systematic review were based on the Preferred Reporting Items of Systematic Reviews and Meta‐Analysis (PRISMA) method (Moher et al., 2009). The protocol of this meta‐analysis was registered in the PROSPERO database with registration code: CRD42023470302.

### 2.1 Search strategy

To find relevant studies, Web of Science, Medline, and Scopus databases were comprehensively searched until September 2023. This search was designed using the PICOS method framework (Participant: adults, Intervention: raspberry consumption, Comparison: control group, Outcome: lipid profile and blood pressure, Type of study: Randomized controlled trials (RCTs)) (Methley et al., 2014).

The search strategy consisted of the following MeSH (Medical Subject Headings) and non‐MeSH terms: (“Raspberry” OR “*Rubus occidentalis*” OR “rubus idaeus” OR “rubus coreanus”) AND (“lipid profile” OR “TG” OR “Triglyceride” OR “Low‐density lipoprotein” OR “LDL” OR “HDL” OR “High density lipoprotein” OR “TC” OR “total cholesterol” OR **“blood pressure” OR “SBP” OR “DBP” OR “systolic blood pressure” OR “diastolic blood pressure”)** AND (“randomized” OR “placebo” OR “clinical trials” OR “randomly” OR “trial” OR “randomized controlled trial” OR “RCT”).

Two researchers independently (M.Sh.J and H.B) screened the found studies based on their titles and abstracts. The reference of the final related articles was checked to reduce the possibility of missing related studies. Also, the Google Scholar search engine was searched manually.

### 2.2 Study selection

The inclusion criteria for this review included (a) interventional studies, (b) raspberry consumption, (c) RCTs, and (d) adult participants.

Animal interventions, non‐interventional studies (observational studies, review articles, short communication, letters to the editors), absence of a control group, lack of reporting related outcomes, and conducting studies in people under 18 years of age were the exclusion criteria of this systematic review.

### 2.3 Data extraction

Data related to this review were independently extracted by two authors (H.B and M.R). Relevant information includes the first author's name, study country, publication date, study design, sample size and number of subjects in each group, characteristics of participants (gender, mean age, mean BMI, and health status), type and duration of intervention with raspberry, type of the control group, and the mean difference and the standard deviation (SD) of the outcomes during the intervention with raspberry (or the mean levels of the outcomes and the standard deviation at the beginning and the end of the intervention). The cases of disagreement were discussed until reaching a consensus.

### 2.4 Quality assessment

The quality of the studies was assessed by two researchers (M.Sh.J and H.B) independently using the Cochrane Collaboration risk of bias tool (Higgins & Green, 2010). This tool assessed the risk of bias in seven subclasses including random sequence generation, allocation concealment, selective reporting, incomplete outcome data, blinding of participants and personnel, blinding of outcome assessor, and other potential sources of bias in three levels: low, unclear, and high. If the number of high‐risk bias items for each study was less than 2, it is considered as general low risk of bias; if it was 2, it is considered as moderate, and if it was greater than 2, it is considered as general high risk of bias. Disagreements were discussed in consultation with a third author (M.M).

### 2.5 Statistical analysis

The overall effect of raspberry intake on lipid markers and blood pressure was estimated by employing the weighted mean differences (WMD) and 95% confidence interval (95%CI) in each group based on the random effects model (DerSimonian & Laird, 1986).

If the mean changes were not reported, it was calculated by subtracting the parameters' mean levels at the interventions beginning from the end. SD changes were estimated using the following formula: SD change = square root [(SDbaseline)^2^ + (SDfinal)^2^ − (2 × R × SDbaseline × SDfinal)] (Borenstein et al., 2021).

The standard error (SEs), interquartile range (IQRs), and 95%CI were converted to SDs using the method of Hozo et al. (2005). The units of all reported lipid markers (TC, TG, LDL‐C, and HDL‐C) were converted to mg/dL and blood pressure to mmHg. Heterogeneity between studies was evaluated by performing Cochran's *Q* test and the measure of the *I*‐square (*I* ^2^) statistic (Higgins et al., 2003). *I* ^2^ > 50% or *p*‐value <.05 was considered a significant heterogeneity between studies (Brondani et al., 2014).

Subgroup analysis to identify the source of heterogeneity based on predetermined criteria (Higgins & Thompson, 2002), including country (Korea and None‐Korea), age (≥35 and <35), gender (both sexes, males, and females), study design (parallel and crossover), baseline BMI (normal, overweight, and obese), health status, duration of intervention (≤8 and >8 weeks), type of intervention (black raspberry and none‐black raspberry), general risk of bias (low and moderate), and outcome values at baseline. Egger's regression and visual interpretation of the funnel plots were used to check the publication bias (Egger et al., 1997). Sensitivity analysis was conducted to investigate the influence of the sample size and quality of each study on the outcomes' pooled effect sizes using the leave‐one‐out method (Duval, 2005; Tobias, 1999). All the analyses performed in this meta‐analysis were performed using Stata version 17 software, and the *p*‐values <.05 were considered statistically significant.

### 2.6 Certainty assessment

The quality of certainty of the studies investigating the effect of raspberry consumption on lipid profile and blood pressure included in this review was evaluated using the GRADE guideline (Grading of Recommendations Assessment, Development, and Evaluation) (Guyatt et al., 2008). The certainty quality of the evidence used for each of the five areas (Risk of bias, Publication Bias, Inconsistency, Indirectness, and Imprecision) was classified into three groups: no serious limitation, serious limitation, and very serious limitation. The overall quality of the evidence was leveled into four: high, moderate, low, and very low.

## 3 RESULTS

### 3.1 Study selection

**FIGURE 1:**
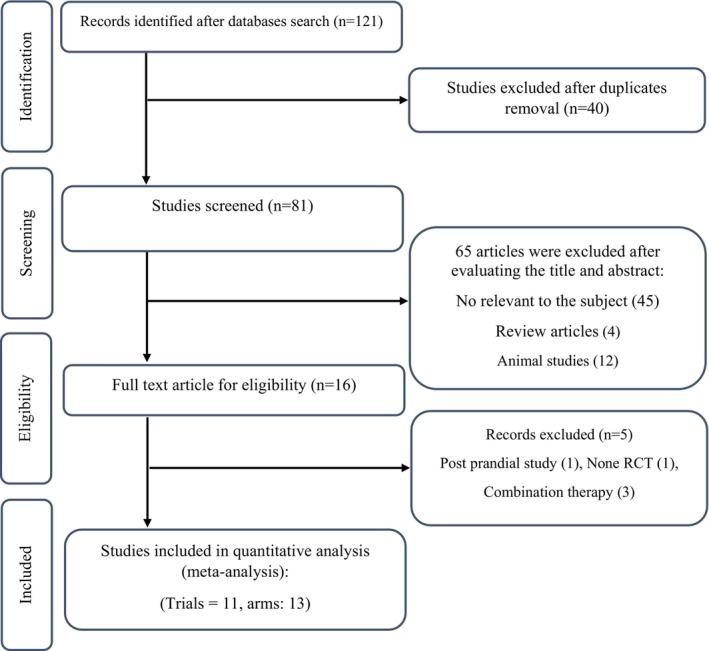
Flowchart of study selection for inclusion trials in this meta‐analysis.

Among the 121 studies found by the initial search, 40 duplicate results were removed. The remaining 81 research were used to be screened. For evaluating the 16 studies, it was necessary to read their full text, which led to the exclusion of five studies due to having no RCT design (*n* = 1), combination therapy (*n* = 3), and lack of a control group (*n* = 1). Finally, 11 studies (13 arms) with 512 participants were eligible to be included in this meta‐analysis (An et al., 2016; Cho et al., 2020; Franck et al., 2020; Franck et al., 2022; Jeong, Hong, et al., 2016; Jeong et al., 2014; Jeong, Kim, et al., 2016; Jung et al., 2023; Mosah et al., 2015; Park et al., 2015; Schell et al., 2019) (Figure 1).

### 3.2 Study characteristics

The studies included in this study were published between 2014 (Jeong et al., 2014) and 2023 (Jung et al., 2023). The study countries included Korea (An et al., 2016; Cho et al., 2020; Jeong, Hong, et al., 2016; Jeong et al., 2014; Jeong, Kim, et al., 2016; Jung et al., 2023; Park et al., 2015), Iraq (Mosah et al., 2015), Canada (Franck et al., 2020, 2022), and the USA (Schell et al., 2019). The study design was crossover in one study (Schell et al., 2019) and parallel in the rest. The intervention was conducted in eight studies on both sexes, and exclusively in two studies on men (Jung et al., 2023; Park et al., 2015), and one on women (Mosah et al., 2015). The sample size of the included studies varied from 22 (Schell et al., 2019) to 77 participants (Cho et al., 2020). The intervention subjects' mean age ranged from 24.65 (Park et al., 2015) to 63.8 years (Jung et al., 2023), and the mean BMI ranged from 23.52 (Cho et al., 2020) to 35.3 kg/m^2^ (Schell et al., 2019). Interventions were performed on healthy people (Park et al., 2015), and individuals with borderline‐high cholesterol levels (Cho et al., 2020), prediabetes (An et al., 2016), type 2 diabetes (Schell et al., 2019), andropause symptoms (Jung et al., 2023), prehypertension (Jeong, Hong, et al., 2016), obesity (Mosah et al., 2015), metabolic syndrome (Jeong et al., 2014; Jeong, Kim, et al., 2016), and at risk of metabolic syndrome (Franck et al., 2020, 2022). The type of raspberry received was black raspberry in seven included studies (An et al., 2016; Cho et al., 2020; Jeong, Hong, et al., 2016; Jeong et al., 2014; Jeong, Kim, et al., 2016; Jung et al., 2023; Park et al., 2015), red raspberry in one (Schell et al., 2019), and raspberry ketones in one study (Mosah et al., 2015), while raspberry type was not reported in two studies (Franck et al., 2020, 2022). The duration of the intervention varied from 4 (Park et al., 2015; Schell et al., 2019) to 12 weeks (An et al., 2016; Cho et al., 2020; Jeong et al., 2014; Jeong, Kim, et al., 2016; Jung et al., 2023; Mosah et al., 2015). A summary of the characteristics of the studies included in this systematic review is shown in Table 1.

**TABLE 1:** Characteristic of included studies in meta‐analysis.

Studies	Country	Study design	Participant	Sample size and sex	Sample size		Trial duration (week)	Means age		Means BMI		Intervention		Main outcomes
					IG	CG		IG	CG	IG	CG	Raspberries dose (mg/d)	Control group	
Jeong et al. (2014)	Korea	Parallel, R, PC, DB	Metabolic Syndrome	73 M & F	38	35	12	58.0 ± 9.2	60.1 ± 9.5	26.3 ± 4.3	25.1 ± 4.0	Black raspberry (*Rubus occidentalis*) extract 750 mg/d	Placebo	TC, TG, LDL‐C, HDL‐C
Park et al. (2015)	Korea	Parallel, R, PC, DB	Healthy smokers	39 M	20	19	4	24.5 ± 2.6	24.8 ± 2.9	NR	NR	Freeze‐dried black raspberry (Rubus coreanus) 30,000 mg/d	Placebo	TC, TG, LDL‐C, HDL‐C, SBP, DBP
Mosah et al. (2015)	Iraq	Parallel, R, PC, SB	Obese women	38 F	20	18	12	31.75 ± 5.58	32.72 ± 7.00	35.41 ± 3.34	34.83 ± 2.99	Raspberry ketones 500 mg/d	Without treatment	TC, TG, LDL‐C, HDL‐C
An et al. (2016)	Korea	Parallel, R, PC, DB	Prediabetes	24 M & F	12	12	12	60.2 ± 8.6	58.4 ± 8.3	24.4 ± 2.3	24.4 ± 1.9	Low‐dose black raspberry extract (*Rubus occidentalis*) 900 mg/d	Placebo	TC, TG, LDL‐C, HDL‐C
An et al. (2016)	Korea	Parallel, R, PC, DB	Prediabetes	27 M & F	15	12	12	58.4 ± 7.4	58.4 ± 8.3	25.0 ± 2.1	24.4 ± 1.9	High‐dose black raspberry extract (*Rubus occidentalis*) 1800 mg/d	Placebo	TC, TG, LDL‐C, HDL‐C
Jeong, Hong, et al. (2016)	Korea	Parallel, R, PC, DB	Prehypertension	30 M & F	15	15	8	60.2 ± 11.2	55.9 ± 12.8	24.5 ± 2.9	25.8 ± 3.0	Moderate‐dose Black raspberry (*Rubus occidentalis*) dried powder extract 1500 mg/d	Placebo	SBP, DBP
Jeong, Hong, et al. (2016)	Korea	Parallel, R, PC, DB	Prehypertension	30 M & F	15	15	8	55.5 ± 12.3	55.9 ± 12.8	23.5 ± 2.4	25.8 ± 3.0	High‐dose black raspberry (*Rubus occidentalis*) dried powder extract 2500 mg/d	Placebo	SBP, DBP
Jeong, Kim, et al. (2016)	Korea	Parallel, R, PC, DB	Metabolic Syndrome	50 M & F	25	25	12	56.4 ± 9.2	60.7 ± 10.4	25.9 ± 4.6	24.7 ± 3.9	Black raspberry (*Rubus occidentalis*) extract 750 mg/d	Placebo	SBP, DBP
Schell et al. (2019)	USA	Crossover, R, C	Type 2 Diabetes	22 M & F	22	22	4	54 ± 19.69	54 ± 19.69	35.3 ± 9.38	35.3 ± 9.38	Frozen red raspberries 250,000 mg/d	Maintained their usual diet	TG, SBP, DBP
Franck et al. (2020)	Canada	Parallel, R, C	At Risk of Metabolic Syndrome	48 M & F	24	24	8	32.46 ± 10.12	31.92 ± 8.05	30.42 ± 5.00	29.38 ± 3.94	Frozen raspberries 280,000 mg/d	Maintained their health and food habits	TC, TG, LDL‐C, HDL‐C, SBP, DBP
Cho et al. (2020)	Korea	Parallel, R, PC, DB	Borderline‐high cholesterol levels	77 M & F	39	38	12	47.03 ± 12.30	47.61 ± 12.20	23.47 ± 2.99	23.58 ± 3.26	Unripe black raspberry (Rubus croreanus) extract	Placebo	TC, TG, LDL‐C, SBP, DBP
Franck et al. (2022)	Canada	Parallel, R, C	Overweight or abdominal obesity, and with slight hyperinsulinemia or hypertriglyceridemia	24 M & F	13	11	8	32.6 ± 10.5	34.0 ± 9.5	29.2 ± 3.9	32.8 ± 5.7	Frozen raspberries 280,000 mg/d	Maintained their usual diet	TC, TG, LDL‐C, HDL‐C, SBP, DBP
Jung et al. (2023)	Korea	Parallel, R, PC, DB	Men with andropause symptoms	30 M	15	15	12	66.13 ± 6.16	61.47 ± 7.65	24.9 ± 2.0	25.8 ± 2.0	Unripe Black Raspberry Extract	Placebo	TC, TG, LDL‐C, HDL‐C

Abbreviations: C, controlled; CG, control group; DB, double‐blinded; DBP, diastolic blood pressure; F, Female; HDL, high‐density lipoprotein; IG, intervention group; LDL, low‐density lipoprotein; M, Male; NR, not reported; PC, placebo‐controlled; R, randomized; SB, single‐blinded; SBP, systolic blood pressure; TC, total cholesterol; TG, Triglycerides.

The general risk of bias of Mosah et al. (2015) was considered moderate, while the rest of the general risk of bias was low. Also, the majority of the included studies were deemed to be of good quality, although the studies conducted by Franck et al. (2022), and Mosah et al. (2015) were assessed as being of fair quality. The details of the risk of bias assessment in each subclass are presented in Table 2.

**TABLE 2:** Risk of bias assessment.

Study	Random sequence generation	Allocation concealment	Selective reporting	Other sources of bias	Blinding (participants and personnel)	Blinding (outcome assessment)	Incomplete outcome data	General risk of bias
Jeong et al. (2014)	L	L	L	U	L	U	L	Low
Park et al. (2015)	U	L	L	L	U	U	L	Low
Mosah et al. (2015)	U	U	L	U	H	H	L	Moderate
An et al. (2016)	L	L	L	U	L	U	L	Low
Jeong, Hong, et al. (2016)	L	U	L	U	L	U	L	Low
Jeong, Kim, et al. (2016)	L	L	L	U	L	U	L	Low
Schell et al. (2019)	U	L	L	L	H	U	L	Low
Franck et al. (2020)	L	L	L	L	H	U	L	Low
Cho et al. (2020)	L	L	L	L	L	U	L	Low
Franck et al. (2022)	U	U	L	U	H	U	L	Low
Jung et al. (2023)	L	U	L	L	L	U	L	Low

*Note*: General Low risk <2 high risk, General moderate risk = 2 high risk, General high risk >2 high risk.

Abbreviations: L, Low; H, High; U, Unclear.

### 3.3 Adverse events

Among the included studies, five did not report the adverse effects that occurred (Franck et al., 2022; Jeong et al., 2014; Mosah et al., 2015; Park et al., 2015; Schell et al., 2019), no adverse events occurred in four studies (Cho et al., 2020; Franck et al., 2020; Jeong, Hong, et al., 2016; Jeong, Kim, et al., 2016), and complications were reported in only two studies (An et al., 2016; Jung et al., 2023). In An et al. (2016), the side effects in the group that received raspberry with a high dose included insomnia, nausea, and skin rash, and in the group that received raspberry with a low dose, it included diarrhea, nausea, abdominal pain, skin rash, and fever. In this study, complaints of fatigue, skin rash, and abdominal pain were reported even in the placebo control group. In another study conducted by Jung et al. (2023), although the relationship between intervention and complaint was ruled out, complications including oral leukoplakia, heartburn, elevated T‐PSA, upper respiratory infection, and dysuria were reported in the group receiving black raspberry extract.

### 3.4 Meta‐analysis

#### 3.4.1 Effect of raspberry consumption on serum TC levels

**FIGURE 2:**
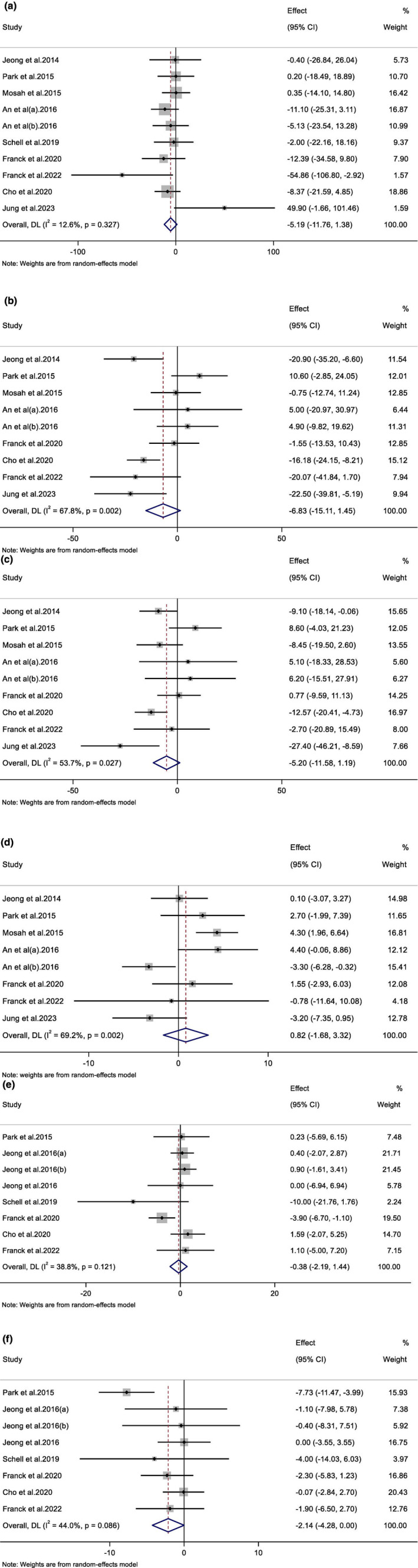
Forest plot detailing weighted mean difference and 95% confidence intervals (CIs) for the effect of Raspberry intake on (a) TG (mg/dL); (b) TC (mg/dL); (c) LDL (mg/dL); (d) HDL (mg/dL); (e) SBP (mmHg); and (f) DBP (mmHg).

Pooling of nine effect sizes showed a non‐significant effect of raspberry consumption on serum total cholesterol levels (WMD, −6.83 mg/dL; 95%CI, (−15.11 to 1.44); *p* = .10; 380 participants). However, significant heterogeneity was observed among the included studies (*I* ^2^ = 67.8%; *p* = .002) (Figure 2a). The subgroup analysis, which was conducted to find the source of heterogeneity, showed a significant reducing effect of raspberry consumption on total cholesterol levels in participants with metabolic syndrome, borderline‐high cholesterol, andropause symptoms, overweight, older than 35 years, and with the baseline elevated serum cholesterol levels (>200 mg/dL) (Table 3).

**TABLE 3:** Subgroup analyses of raspberry consumption on lipid profile and blood pressure in adults.

	NO	WMD (95%CI)	*p*‐value	Heterogeneity		
				*p* heterogeneity	*I* ^2^	*p* between sub‐groups
Subgroup analyses of raspberry consumption on TC (mg/dL)						
Overall effect	9	−6.83 (−15.11, 1.44)	.10	.002	67.8%	
Country						
Korea	6	−7.25 (−19.09, 4.59)	.23	.001	75.9%	.68
None‐Korea	3	−4.13 (−13.26, 4.99)	.37	.28	20.9%	
Trial duration (week)						
≤8	3	−1.65 (−16.43,13.12(	.82	.05	65.0%	.38
>8	6	−9.48 (−18.99, 0.03)	.05	.01	64.0%	
Intervention						
Black raspberry	6	−7.25 (−19.09, 4.59)	.23	.001	75.9%	.68
None‐black raspberry	3	−4.13 (−13.26, 4.99)	.37	.28	20.9%	
Health status						
METS	1	−20.90 (−35.19, −6.60)	**.004**	‐	‐	**.003**
Healthy	2	4.50 (−6.59, 15.59)	.42	.21	34.4%	
Prediabetes	2	4.92 (−7.88, 17.72)	.45	.99	0.0%	
At risk of METS	2	−8.48 (−26.05, 9.08)	.34	.14	53.1%	
Borderline‐high cholesterol	1	−16.18 (−24.14, −8.21)	**<.001**	–	–	
Andropause symptoms	1	−22.50 (−39.81, −5.18)	**.01**	–	–	
Age						
≤35	4	−0.79 (−10.51, 8.93)	.87	.12	47.6%	.14
>35	5	−11.57 (−22.16, −0.98)	**.03**	.03	61.7%	
Gender						
Both sexes	6	−8.98 (−18.27, 0.30)	.05	.02	59.8%	.56
Males	2	−5.48 (−37.90, 26.94)	.74	.003	88.6%	
Females	1	−0.75 (−12.74, 11.24)	.90	‐	‐	
Baseline BMI						
Normal (18.5–24.9)	4	−0.005 (−15.56, 15.55)	1.000	.002	79.9%	**.02**
Overweight (25–29.9)	2	−21.54 (−32.57, −10.52)	**<.001**	.88	0.0%	
Obese (>30)	3	−4.13 (−13.26, 4.99)	.37	.28	20.9%	
Baseline TC (mg/dL)						
≤200	8	−5.16 (−14.09, 3.76)	.25	.009	62.5%	.07
>200	1	−16.18 (−24.14, −8.21)	**<.001**	–	–	
General risk of bias						
Low	8	−7.72 (−17.04, 1.60)	.10	.002	69.9%	.36
Moderate	1	−0.75 (−12.74, 11.24)	.90	–	–	
Subgroup analyses of raspberry consumption on TG (mg/dL)						
Overall effect	10	−5.19 (−11.76, 1.37)	.12	.32	12.6%	
Country						
Korea	6	−5.00 (−13.12, 3.11)	.22	.33	12.5%	.80
None‐Korea	4	−6.99 (−20.66, 6.67)	.31	.20	34.5%	
Trial duration (week)						
≤8	4	−7.75 (−21.98, 6.47)	.28	.23	3.0%	.69
>8	6	−4.48 (−12.40, 3.43)	.26	.31	16.0%	
Intervention						
Black raspberry	6	−5.00 (−13.12, 3.11)	.22	.33	12.5%	.80
None‐black raspberry	4	−6.99 (−20.66, 6.67)	.31	.30	34.5%	
Health status						
METS	1	−0.40 (−26.83, 26.03)	.97	–	–	.29
Prediabetes	2	−8.87 (−20.11, 2.37)	.12	.61	.0%	
Diabetes type 2	1	−2.00 (−22.15, 18.15)	.84	–	–	
Healthy	2	0.29 (−11.13, 11.72)	.96	.99	0.0%	
At risk of METS	2	−26.86 (−66.32, 12.58)	.18	.14	54.0%	
Borderline‐high cholesterol	1	−8.37 (−21.59, 4.85)	.21	–	–	
Andropause symptoms	1	49.90 (−1.66, 101.46)	.05	–	–	
Age						
≤35	4	−6.30 (−19.98, 7.38)	.36	.36	8.5%	.92
>35	6	−5.56 (−13.54, 2.40)	.17	.18	37.1%	
Gender						
Both sexes	7	−8.48 (−15.57, −1.39)	**.01**	.65	.0%	.32
Males	2	19.00 (−28.23, 66.24)	.43	.07	68.3%	
Females	1	0.35 (−14.10, 14.80)	.96	–	–	
Baseline BMI						
Normal (18.5–24.9)	4	−7.12 (−14.91, 0.66)	.07	.80	0.0%	.56
Overweight (25–29.9)	2	19.67 (−28.60, 67.95)	.42	.08	65.5%	
Obese (>30)	4	−6.99 (−20.66, 6.67)	.31	.20	34.5%	
Baseline TG (mg/dL)						
≤150	7	−6.62 (−13.33, 0.09)	.05	.46	0.0%	.36
>150	3	3.32 (−17.24, 23.90)	.75	.14	49.1%	
General risk of bias						
Low	9	−6.20 (−13.67, 1.26)	.10	.29	16.6%	.43
Moderate	1	0.35 (−14.10, 14.80)	.96	–	–	
Study design						
Parallel	9	−5.46 (−12.86, 1.93)	.14	.25	21.4%	.75
Crossover	1	−2.00 (−22.15, 18.15)	.84	–	–	
Subgroup analyses of raspberry consumption on LDL (mg/dL)						
Overall effect	9	−5.19 (−11.58, 1.18)	.11	.02	53.7%	
Country						
Korea	6	−5.81 (−15.38, 3.75)	.23	.01	66.5%	.69
None‐Korea	3	−3.41 (−10.39, 3.56)	.33	.48	.0%	
Trial duration (week)						
≤8	3	2.84 (−4.48, 10.17)	.44	.52	0.0%	.01
>8	6	−9.65 (−16.11, −3.19)	**.003**	.18	33.7%	
Intervention						
Black raspberry	6	−5.81 (−15.38, 3.75)	.23	.01	66.5%	.69
None‐black raspberry	3	−3.41 (−10.39, 3.56)	.33	.48	.0%	
Health status						
METS	1	−9.10 (−18.13, −0.06)	**.04**	–	–	.03
Healthy	2	−0.21 (−16.91, 16.48)	.98	.04	74.8%	
Prediabetes	2	5.69 (−10.23, 21.61)	.48	.94	0.0%	
At risk of METS	2	−0.07 (−9.08, 8.92)	.98	.74	0.0%	
Borderline‐high cholesterol	1	−12.57 (−20.40, −4.73)	**.002**	–	–	
Andropause symptoms	1	−27.40 (−46.20, −8.59)	**.004**	–	–	
Age (year)						
≤35	4	−0.51 (−7.79, 6.76)	.88	.25	26.7%	.10
>35	5	−9.66 (−18.07, −1.24)	**.02**	.11	46.2%	
Gender						
Both sexes	6	−5.50 (−11.52, 0.50)	.07	.22	27.9%	.89
Males	2	−8.69 (−43.95, 26.55)	.62	.002	89.7%	
Females	1	−8.45 (−19.49, 2.59)	.13	–	–	
Baseline BMI						
Normal (18.5–24.9)	4	0.26 (−13.10, 13.63)	.96	.02	69.3%	**.31**
Overweight (25–29.9)	2	−16.31 (−33.84, 1.21)	.06	.08	66.2%	
Obese (>30)	3	−3.41 (−10.39, 3.56)	.33	.48	0.0%	
Baseline LDL‐C (mg/dL)						
≤130	8	−3.68 (−10.72, 3.35)	.30	.05	49.6%	.09
>130	1	−12.57 (−20.40, −4.73)	**.002**	–	–	
General risk of bias						
Low	8	−4.58 (−11.98, 2.81)	.22	.01	59.1%	.56
Moderate	1	−8.45 (−19.49, 2.59)	.13	–	–	
Subgroup analyses of raspberry consumption on HDL (mg/dL)						
Overall effect	8	0.82 (−1.67, 3.32)	.51	.002	69.2%	
Country						
Korea	5	−0.10 (−2.99, 2.78)	.94	.02	65.4%	.04
None‐Korea	3	3.55 (1.51, 5.59)	**.001**	.41	0.0%	
Trial duration (week)						
≤8	3	1.86 (−1.24, 4.96)	.23	.83	0.0%	.55
>8	5	0.47 (−2.97, 3.92)	.78	<.001	81.9%	
Intervention						
Black raspberry	5	−0.10 (−2.99, 2.78)	.94	**.02**	65.4%	.04
None‐black raspberry	3	3.55 (1.51, 5.59)	**.001**	.41	0.0%	
Health status						
METS	1	0.10 (−3.07, 3.27)	.95	–	–	.02
Healthy	2	3.98 (1.88, 6.07)	**<.001**	.55	0.0%	
Prediabetes	2	0.36 (−7.17, 7.90)	.92	.005	87.4%	
At Risk of METS	2	1.21 (−2.93, 5.35)	.56	.69	0.0%	
Andropause symptoms	1	−3.20 (−7.35, 0.95)	.13	–	–	
Age						
≤35	4	3.41 (1.54, 5.28)	**<.001**	.59	0.0%	.03
>35	4	−0.68 (−3.90, 2.53)	.67	.02	68.3%	
Gender						
Both sexes	5	0.26 (−2.59, 3.12)	.85	.06	54.9%	.06
Males	2	−0.35 (−6.13, 5.42)	.90	.06	70.7%	
Females	1	4.30 (1.95, 6.64)	**<.001**	–	–	
Baseline BMI						
Normal (18.5–24.9)	3	1.02 (−4.07, 6.12)	.69	.008	79.4%	.04
Overweight (25–29.9)	2	−1.26 (−4.45, 1.91)	.43	.21	34.8%	
Obese (>30)	3	3.55 (1.51, 5.59(	**.001**	.41	0.0%	
Baseline HDL (mg/dL)						
≤50	4	0.56 (−3.92, 5.05)	.80	<.001	86.2%	.86
>50	4	1.00 (−1.21, 3.21)	.37	.80	0.0%	
General risk of bias						
Low	7	0.04 (−2.27, 2.37)	.96	.05	51.3%	.01
Moderate	1	4.30 (1.95, 6.64)	**<.001**	–	–	
Subgroup analyses of raspberry consumption on SBP (mm Hg)						
Overall effect	8	−0.37 (−2.19, 1.44)	.68	.12	38.8%	
Country						
Korea	5	0.74 (−0.74, 2.24)	.32	.98	0.0%	.10
None‐Korea	3	−3.08 (−7.45, 1.28)	.16	.18	41.6%	
Trial duration (week)						
≤8	6	−0.80 (−3.07, 1.47)	.48	.07	50.9%	.31
>8	2	1.24 (−1.99, 4.47)	.45	.69	.0%	
Intervention						
Black raspberry	5	0.74 (−0.74, 2.24)	.32	.98	0.0%	.10
None‐black raspberry	3	−3.08 (−7.45, 1.28)	.16	.18	41.6%	
Health status						
METS	1	0.00 (−6.93, 6.93)	1.000	–	–	.46
Healthy	1	0.23 (−5.69, 6.15)	.93	–	–	
Prehypertension	2	0.64 (−1.11, 2.40)	.47	.78	0.0%	
T2DM	1	−10.00 (−21.75, 1.75)	.09	–	–	
At Risk of METS	2	−2.16 (−6.82, 2.50)	.36	.14	53.2%	
Borderline‐high cholesterol	1	1.59 (−2.06, 5.24)	.39	–	–	
Age						
≤35	3	−1.80 (−5.16, 1.55)	.29	.21	35.9%	.20
>35	5	0.60 (−0.93, 2.13)	.44	.47	0.0%	
Gender						
Both sexes	7	−0.43 (−2.46, 1.58)	.67	.07	47.3%	.83
Males	1	0.23 (−5.69, 6.15)	.93	–	–	
Baseline BMI						
Normal (18.5–24.9)	5	0.74 (−0.74, 2.24)	.32	.98	0.0%	.10
Obese (>30)	3	−3.08 (−7.45, 1.28)	.16	.18	41.6%	
Study design						
Parallel	7	−0.17 (−1.85, 1.49)	.83	.18	31.9%	.10
Crossover	1	−10.00 (−21.75, 1.75)	.09	–	–	
Baseline SBP (mm Hg)						
≤130	5	−0.68 (−3.37, 2.00)	.61	.15	40.3%	.63
>130	3	0.19 (−2.29, 2.68)	.87	.20	36.7%	
Subgroup analyses of raspberry consumption on DBP (mm Hg)						
Overall effect	8	−2.14 (−4.27, 0.00)	.05	.08	44.0%	
Country						
Korea	5	−2.02 (−5.46, 1.40)	.24	.01	67.4%	.90
None‐Korea	3	−2.28 (−4.98, 0.41)	.09	.93	0.0%	
Trial duration (week)						
≤8	6	−3.44 (−5.95, −0.93)	**.007**	0.23	27.2%	.04
>8	2	−0.04 (−2.22, 2.14)	0.96	0.97	0.0%	
Intervention						
Black raspberry	5	−2.02 (−5.46, 1.40)	0.24	.01	67.4%	.90
None‐black raspberry	3	−2.28 (−4.98, 0.41)	.09	0.93	0.0%	
Health status						
METS	1	0.00 (−3.55, 3.55)	1.000	–	–	.02
Prehypertension	2	−0.79 (−5.99, 4.39)	0.76	0.89	0.0%	
Healthy	1	−7.73 (−11.47, −3.98)	**<.001**	–	–	
T2DM	1	−4.00 (−14.03, 6.03)	.43	–	–	
At Risk of METS	2	−2.15 (−4.95, 0.64)	.13	.89	0.0%	
Borderline‐high cholesterol	1	−0.07 (−2.84, 2.70)	.96	–	–	
Age						
≤35	3	−4.06 (−7.82, −0.29)	.03	.06	63.6%	.08
>35	5	−0.30 (−2.28, 1.66)	.76	.96	0.0%	
Gender						
Both sexes	7	−0.91 (−2.53, 0.69)	.26	.94	0.0%	.001
Males	1	−7.73 (−11.47, −3.98)	**<.001**	–	–	
Baseline BMI						
Normal (18.5–24.9)	5	−2.02 (−5.46, 1.40)	.24	.01	67.4%	.90
Obese (>30)	3	−2.28 (−4.98, 0.41)	.09	.93		
Study design						
Parallel	7	−2.06 (−4.34, 0.22)	.07	.05	51.3%	.71
Crossover	1	−4.00 (−14.03, 6.03)	.43	–	–0.0%	
Baseline DBP						
≤80	5	−2.31 (−5.08, 0.46)	.10	.01	67.0%	.76
>80	3	−1.47 (−6.08, 3.13)	.53	.85	0.0%	

*Note*: Bold indicates statistical significance value (*p* < .05).

Abbreviations: BMI, Body Mass Index; CI, confidence interval; Crossover, R, PC, DB, Crossover, randomized placebo‐controlled double‐blind; DBP, diastolic blood pressure; HDL, high‐density lipoprotein; LDL, low‐density lipoprotein; METS, metabolic syndrome; parallel, R, PC, DB, parallel, randomized placebo‐controlled double‐blind; parallel, R, PC, SB, parallel, randomized placebo‐controlled single‐blind; SBP, systolic blood pressure; TC, total cholesterol; TG, Triglycerides; WMD, weighted mean differences.

#### 3.4.2 Effect of raspberry consumption on serum TG levels

Meta‐analyzing 10 effect sizes showed that raspberry consumption could not significantly change serum TG levels compared to control groups (WMD, −5.19 mg/dL; 95%CI, (−11.76 to 1.37); *p* = .12; 402 participants). Also, heterogeneity among the included studies was not significant (*I* ^2^ = 12.6%; *p* = .32) (Figure 2b). Subgroup analysis indicated that raspberry intake significantly decreased serum TG levels in studies conducted on both sexes (Table 3).

#### 3.4.3 Effect of raspberry consumption on serum LDL‐C levels

The combination of nine effect sizes showed that receiving raspberry had no significant effect on serum LDL‐C levels (WMD, −5.19 mg/dL; 95%CI, (−11.58 to 1.18); *p* = .11; 380 participants). While heterogeneity between studies was significant (*I* ^2^ = 53.7%; *p* = .02) (Figure 2c), the subgroup analysis reported the significant effect of raspberry intake on LDL reduction in studies with more than 8‐week duration, and those conducted on participants with metabolic syndrome, borderline‐high cholesterol, andropause symptoms, overweight, and more than 35 years (Table 3).

#### 3.4.4 Effect of raspberry consumption on serum HDL‐C levels

The combination of eight effect sizes demonstrated a non‐significant effect of raspberry consumption on serum HDL‐C levels (WMD, 0.82 mg/dL; 95%CI, (−1.67 to 3.32); *p* = .51; 303 participants). However, significant heterogeneity was detected among the included studies (*I* ^2^ = 69.2%; *p* = .002) (Figure 2d). The significant increasing effect of raspberry intake on serum HDL levels in studies conducted in non‐Korean countries, with moderate general risk of bias, interventions with none‐exclusively black raspberries, and in obese, females, 35 years old or younger, and healthy subjects, was reported by subgroup analysis (Table 3).

#### 3.4.5 Effect of raspberry consumption on SBP

After pooling eight effect sizes, the meta‐analysis showed that raspberry consumption had no significant effect on SBP compared to control groups (WMD, −0.37 mm Hg; 95%CI, (−2.19 to 1.44); *p* = .68; 320 participants). Furthermore, heterogeneity among the included studies was not significant (*I* ^2^ = 38.8%; *p* = .12) (Figure 2e).

#### 3.4.6 Effect of raspberry consumption on DBP

**FIGURE 3:**
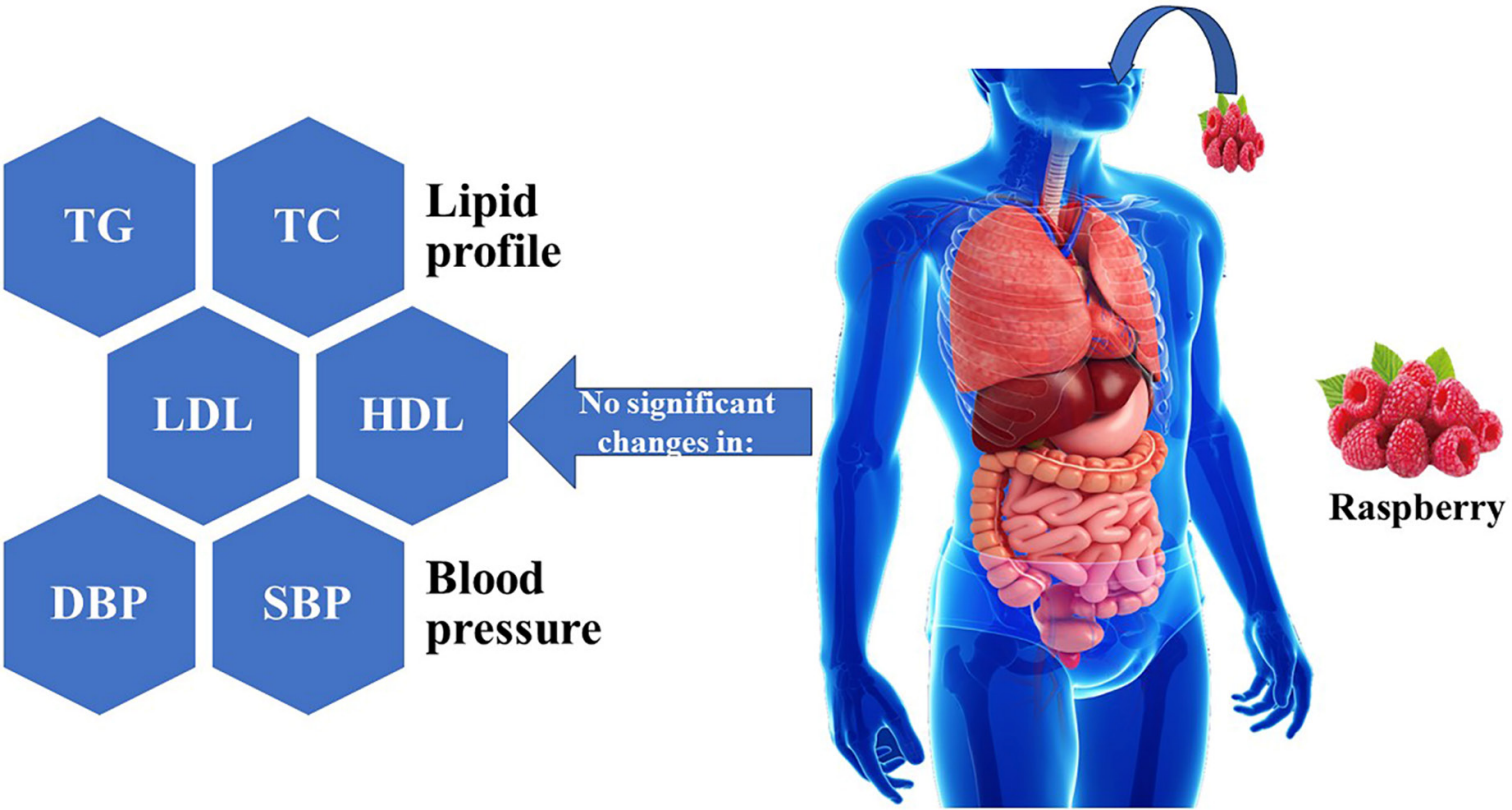
Raspberry consumption can improve the blood pressure and lipid profile but the changes were not significant.

Combining eight effect sizes showed that receiving raspberry did not lead to a significant change in DBP (WMD, −2.14 mm Hg; 95%CI, (−4.27 to 0.00); *p* = .05; 320 participants) (Figure 3). In addition, there was non‐significant heterogeneity among the included studies (*I* ^2^ = 44.0%; *p* = .08) (Figure 2f).

### 3.5 Sensitivity analysis and publication bias

**FIGURE 4:**
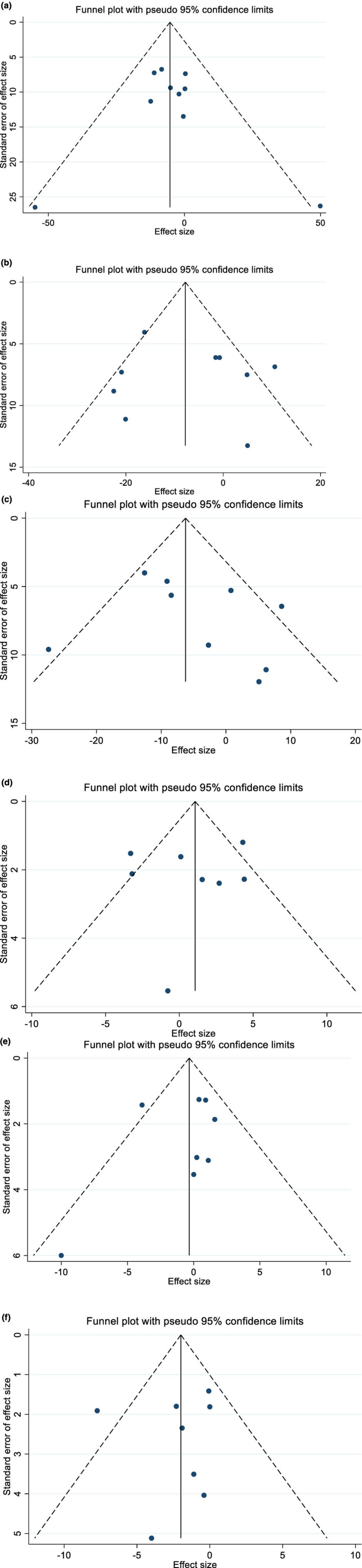
Funnel plots for the effect of Raspberry intake on (a) TG (mg/dL); (b) TC (mg/dL); (c) LDL (mg/dL); (d) HDL (mg/dL); (e) SBP (mmHg); and (f) DBP (mmHg).

Egger's test and visual inspection of the funnel plots showed no significant publication bias in the studies examining the effect of raspberry on total cholesterol (*p* = .63), TG (*p* = .76), LDL‐C (*p* = .43), HDL‐C (*p* = .68), SBP (*p* = .65), and DBP (*p* = .77) (Figure 4a–f). The sensitivity analysis showed that the general result of the effect of raspberry intake on HDL‐C and SBP did not depend on the presence of a specific study. The result of the effect of raspberry intake on TC and TG significantly changed after omitting the study conducted by Park et al. (WMD: −9.23 mg/dL (−17.04, −1.43)) (Park et al., 2015), and Jung et al. (WMD: −6.04 mg/dL (−12.07, −0.02)) (Jung et al., 2023), respectively. Also, the pooled effect sizes for LDL‐C after omitting the effect sizes of Park et al. (WMD: −7.30 mg/dL (−13.14, −1.45)) (Park et al., 2015), and for DBP after removing Jeong et al. (WMD: –2.57 mm Hg (–5.00, –0.13)) (Jeong, Kim, et al., 2016), and Cho et al. (WMD: –2.66 mm Hg (–5.07, –0.26)) (Cho et al., 2020), changed significantly.

### 3.6 GRADE analysis

The assessment of the quality of evidence examined in this meta‐analysis was done based on the GRADE protocol (Guyatt et al., 2008). The certainty of the evidence of the effect of raspberry consumption on total cholesterol, LDL‐C, and HDL‐C was downgraded to low due to serious limitations in inconsistency and imprecision. The evidence used to investigate the effect of raspberry consumption on TG, SBP, and DBP moderate quality was mentioned due to serious limitations in imprecision. The grade profile for the evidence included in this meta‐analysis is shown in Table 4.

**TABLE 4:** GRADE profile of raspberry consumption for lipid profile and blood pressure.

Quality assessment						Summary of findings		Quality of evidence
Outcomes	Risk of bias	Inconsistency	Indirectness	Imprecision	Publication bias	Number of intervention/controls	WMD (95%CI)	
TC	No serious limitations	Serious limitations^a^	No serious limitations	Serious limitations^b^	No serious limitations	196/184	−6.83 mg/dL (−15.11, 1.44)	⊕⊕◯◯ Low
TG	No serious limitations	No serious limitations	No serious limitations	Serious limitations^b^	No serious limitations	218/206	−5.19 mg/dL (−11.76, 1.37)	⊕⊕⊕◯ Moderate
LDL‐C	No serious limitations	Serious limitations^a^	No serious limitations	Serious limitations^b^	No serious limitations	196/184	−5.19 mg/dL (−11.58, 1.18)	⊕⊕◯◯ Low
HDL‐C	No serious limitations	Serious limitations^a^	No serious limitations	Serious limitations^b^	No serious limitations	157/146	0.82 mg/dL (−1.67, 3.32)	⊕⊕◯◯ Low
SBP	No serious limitations	No serious limitations	No serious limitations	Serious limitations^b^	No serious limitations	173/169	−0.37 mm Hg (−2.19, 1.44)	⊕⊕⊕◯ Moderate
DBP	No serious limitations	No serious limitations	No serious limitations	Serious limitations^b^	No serious limitations	173/169	−2.14 mm Hg (−4.27, 0.00)	⊕⊕⊕◯ Moderate

^a^ There is high heterogeneity (*I* ^2^ > 50%).

^b^ There is no evidence of significant effects of raspberry consumption.

## 4 DISCUSSION

The present systematic review and meta‐analysis study with the review of 13 RCTs showed that the consumption of raspberries reduces the serum level of TC in subjects who are overweight, over 35 years old and with high cholesterol level. LDL‐C is reduced in the intervention group for more than 8 weeks with raspberries and in subjects with overweight and aged over 35 years. The level of HDL‐C increases in black raspberry group, women, obese, subjects over 35 years old, and healthy group. Also, the intervention with raspberry has been effective in reducing DBP in less than 8‐week intervention and in men and healthy subjects.

In the meta‐analysis study by Nikparast et al. (2023) published in 2023, the review of six RCTs showed that raspberry and blackcurrant have no effect on lowering blood pressure. In another review (2020) that investigated the effect of berries, including raspberries, on cardiovascular risk factors, no significant effect on the improvement of these factors was seen (Wang et al., 2021). It should be mentioned that in this review, only one RCT that investigated the effect of raspberries on DBP and SBP was included (Jeong, Hong, et al., 2016).

Raspberry, specifically red raspberry (*Rubus idaeus*), has been studied for its potential health benefits, including its role in improving hypertension and lipid profile. The mechanism of action by which raspberry may exert this effect is not fully understood. Some studies have provided insights into its potential mechanisms. The authors highlighted that raspberries are abundant sources of bioactive compounds such as polyphenols, anthocyanins, and dietary fiber, which have been presented to influence lipid metabolism. They suggested that these compounds may modulate enzymes involved in lipid digestion, absorption, and synthesis, ultimately leading to the improvement of lipid profile (Teng et al., 2017). The cholesterol‐lowering effect of raspberry might be attributed to the inhibition of hepatic cholesterol synthesis by inhibiting β‐hydroxy β‐methylglutaryl‐CoA (HMG‐CoA) reductase and increased fecal excretion of bile acids (Wang et al., 2012). Also, upregulate the hepatic LDL‐C receptor expression (Tu et al., 2019).

The researchers found that raspberry ketone treatment significantly reduced intracellular lipid accumulation by inhibiting fatty acid synthesis, promoting fatty acid oxidation, and inhibiting lipogenesis by decreasing the levels of the peroxisome proliferator‐activated receptor γ (PPARγ) mRNA in adipose tissues (Askar et al., 2023). These findings suggest that raspberry ketone may contribute to lowering blood cholesterol levels by modulating lipid metabolism. Raspberry ketone treatment significantly decreased triglyceride accumulation and increased lipolysis. The researchers proposed that these effects were mediated by activating adiponectin signaling, a hormone involved in regulating lipid metabolism (Park, 2010). Moreover, the expression of the CCATT/enhancer‐binding protein α (C/EBPα), sterol regulatory element‐binding protein‐1c (SREBP‐1c), acetyl‐CoA carboxylase (ACC), and fatty acid synthase (FAS) mRNAs was decreased (Oh et al., 2016). Also, phosphorylation of 5′ adenosine monophosphate‐activated protein kinase (AMPK) in the liver increased. This, in turn, led to a decrease in cholesterol biosynthesis due to the inhibition of SREBP‐2 activation (Cho et al., 2020).

As shown in the results of the present meta‐analysis and also mentioned in the first paragraph of the discussion, the effect of raspberry in improving the lipid profile, including the reduction of TC and LDL‐C in people who are exposed to lipid disorders, such as overweight people and over 35 years old. It is more obvious that this shows the regulating effect of raspberry on the lipid profile, and it cannot be concluded that it only reduces the level of TC and LDL‐C. Also, long‐term intervention (more than 8 weeks) is more effective. One of the factors that are effective in increasing the level of HDL‐C is dietary antioxidants. In this meta‐analysis, it was also shown that red raspberry, which is thought to have a higher level of antioxidants, significantly increased the level of HDL‐C. Also, in general, the level of HDL‐C in women is higher than that of men due to hormonal factors, and their response to raspberry was also significant in increasing the level of HDL‐C.

The polyphenolic compounds present in raspberries enhanced nitric oxide production and improved endothelial function (Lee et al., 2014). Nitric oxide helps relax blood vessels, resulting in better blood flow and potentially lower blood pressure. Furthermore, polyphenols inhibited enzymes like endothelin‐1 related to vasoconstriction, enhancing vasodilation, and reducing oxidative stress (Nikparast et al., 2023). Delphinidin‐3‐O‐sambubiosides and cyanidin‐3‐O‐sambubiosides in black raspberry play an effective role in regulating blood pressure through the inhibition of renin‐angiotensin system (Lee et al., 2014). These mechanisms collectively promote a healthier cardiovascular system and may contribute to blood pressure reduction.

Based on the information provided, it seems that the meta‐analysis conducted on the effect of raspberry on lipid profile and blood pressure had some limitations. These include a small number of eligible studies for meta‐analysis and low quality of the most included RCTs. The heterogeneity among the included RCTs was also high.

Furthermore, the sensitivity analysis results for TC, TG, and LDL‐C did not allow for a definite conclusion regarding the effectiveness of raspberry. The grade test indicated moderate‐to‐low‐quality results, suggesting that more and better RCTs are needed in this field to draw a definitive conclusion.

## 5 CONCLUSION

While this study provides some insights into the potential mechanisms underlying raspberry's effects on improving lipid profile and blood pressure, it is critical to consider that further research is needed to fully understand the exact mechanism of action. Additionally, it is worth mentioning that individual responses may vary, and other factors such as diet and lifestyle should also be considered.

## AUTHOR CONTRIBUTIONS

**Mostafa Shahraki Jazinaki:** Conceptualization (equal); formal analysis (equal); investigation (equal); writing – original draft (equal). **Hossein Bahari:** Project administration (equal); writing – review and editing (equal). **Mohammad Rashidmayvan:** Writing – original draft (equal). **Seyyed Mostafa Arabi:** Formal analysis (equal). **Iman Rahnama:** Data curation (equal); investigation (equal). **Mahsa Malekahmadi:** Validation (equal); visualization (equal); writing – original draft (equal); writing – review and editing (equal).

## CONFLICT OF INTEREST STATEMENT

The authors declare that they have no conflict of interest.

## Data Availability

Data will be available upon reasonable request from the authors.
